# Carbonic Anhydrase 4 serves as a Clinicopathological Biomarker for Outcomes and Immune Infiltration in Renal Cell Carcinoma, Lower Grade Glioma, Lung Adenocarcinoma and Uveal Melanoma

**DOI:** 10.7150/jca.46902

**Published:** 2020-08-25

**Authors:** Yue Xu, Wen-Hao Xu, Shen-Nan Shi, Xiao-Long Yang, Ya-Ru Ren, Xin-Yu Zhuang, Yuan-Yuan Qu, Hai-Liang Zhang, Xiao-Feng Zhang

**Affiliations:** 1 Department of Ophthalmology, First Affiliated Hospital of Soochow University, Suzhou, 215000, China; 2 Medical College, Soochow University, Suzhou, 215000, China; 3 Department of Urology, Fudan University Shanghai Cancer Center, Shanghai 200032, China; 4 Cancer Institute, Fudan University Shanghai Cancer Center, Shanghai 200032, China; 5 Department of Oncology, Shanghai Medical College, Fudan University, Shanghai 20032, China

**Keywords:** carbonic anhydrase 4, tumor immune microenvironment, prognosis, immune infiltration, biomarker

## Abstract

**Background:** Carbonic anhydrase 4 (CA4) maintains homeostasis of carbon dioxide and bicarbonate. It is suggested to be a potential prognostic biomarker, while the correlations between *CA4* and different cancers are indistinct.

**Methods:** Differential mRNA expression of *CA4* among different cancers and corresponding normal tissues was compared based on datasets on the Cancer Genome Atlas (TCGA) platforms. Then, survival analysis was performed using Tumor-immune system interactionsplatform and TCGA cohort on the basis of distinct comparison expression of *CA4* in five kinds of tumors. In addition, molecular penal analysis and functional annotations of *CA4*-related genes was elaborated. The correlation between *CA4* mRNA expression and tumor immune microenvironment were analyzed in detail.

**Results:** Compared with adjacent normal tissues, *CA4* mRNA expressions were found significantly lower in various tumors. Moreover, decreased expression of *CA4* was significantly related to worse overall survival (OS) and progression-free survival (PFS) in kidney renal clear cell carcinoma (KIRC), brain lower grade glioma (LGG), lung adenocarcinoma (LUAD) and uveal melanoma (UVM), and worse OS of prostate adenocarcinoma (PRAD) (*p*<0.05). Cox regression analyses indicated that *CA4* was a significant prognostic biomarker in KIRC, LGG, LUAD and UVM. Moreover, *CA4* showed markedly relationship with tumor immune environment and diverse immune infiltration signatures in KIRC, LGG, LUAD and UVM.

**Conclusions:** Our study revealed that* CA4* was a potential biomarker for aggressive progression and poor prognosis in KIRC, LGG, LUAD, PRAD and UVM, correlated with immune infiltration in various tumor environments. These results suggested that *CA4* possibly served as a promising prognostic and immune infiltration biomarker in many cancers.

## Introduction

Tumor microenvironment consists of immune cells, surrounding blood vessels, signaling molecules, fibroblasts and extracellular matrix 1. Tumor immune microenvironment (TIME) is tightly associated with location, density and organization of immune cells and cytokines in diverse tumors 2. The existence of antitumor environment represented by Th1 helper cells, CD8^+^ cytotoxic T cells and correlated cytokines usually implies the extent of TIME and even leads to the elimination of tumors 3. Infiltrating immune cells were highly relevant to heterogeneity of tumor cells in different parts of immunotherapy efficacy 4. The heterogeneity of tumors results in cells secreting distinct molecular markers with diverse levels of sensitivity to treatment 5. Immunotherapeutic therapies were also utilized in many cancers, such as skin melanoma (SKCM), kidney renal clear cell carcinoma (KIRC) and non-small cell lung cancer (NCSLC) 6. Immune checkpoint blockade and targeted therapy has achieved great breakthroughs in melanoma treatment 7. Interleukin 2, immune checkpoint inhibitors and interferon alfa have been applied in KIRC treatment guidelines 8. The outcomes of lung cancer patients are influenced by TIME which balances the suppressive factors and cytotoxic responses 9. Notably, the mechanisms by which tumors evolve to remove host defenses and evade immune control vary from cancer to cancer, and special markers of different tumors are reflected in its microenvironment 10. Therefore, it is of great importance to know the molecular interactions and biomarkers in TIME and may provide novel targets for immune therapies in these tumors.

Carbonic anhydrases (CAs) are zinc metalloenzymes, involved in various biological processes, including tumorigenicity, lipogenesis, gluconeogenesis, ureagenesis and development and toxicity of different pathogens 11. CAs catalyze the fundamental reaction for life, which is the interconversion of water and carbon dioxide into dissociated ions of carbonic acid 12,13. The 14 human isozymes of CAs are up-regulated and down-regulated from gene levels in diverse dysfunctions or diseases, which promotes CAs to be considered as disease biomarkers 14. Representatively, *CA9* is a uniformly induced gene as a reliable biomarker of hypoxia and worse prognosis in tumors, such as prostate and renal cancer 15,16. The higher expression of *CA12* was proved to be a marker of better prognosis in NCSLC 17 and breast cancer 18. Moreover, the invasion of renal carcinoma cells was suggested to be restrained by inhibiting *CA12* 19.

Among the membrane-associated CA isoforms, *CA4* is anchored by glycosylphosphatidylinositol linkage and the most widely distributed 20. *CA4* was found important in bicarbonate reabsorption of kidney 21. It also maintains homeostasis of carbon dioxide and bicarbonate in brain, catalyzes the carbon dioxide exchange and regulates local pH in lung 22. *CA4* was suggested to be a biomarker of diagnosis, progression or immune microenvironment in the following diseases. The disruption of *CA4* may be associated with the perturbation of pH homeostasis in retina and correlated with retinitis pigmentosa 23. In addition, *CA4* markedly inhibited capacity of invasion and migration in colon cancer cells 24. Interestingly, *CA4* was recently reported as an up-regulated differential expressed gene to participate in the TIME of KIRC 24.

In present study, we explored prognostic significance of *CA4* among pan-cancers. Functional annotations and immune infiltration correlation were also investigated between *CA4* and related cancers. TIME correlated with *CA4* was displayed to uncover the underlying molecular mechanisms in these cancers.

## Materials and Methods

### Ethics statement

Study procedures were approved by First Affiliated Hospital of Soochow University (Suzhou, China) included in this research (ID: 2019-076). Written informed consents were acquired from online open-access databases.

### Oncomine database

The transcriptional expression of *CA4* in diverse cancers was obtained from Oncomine database (https://www.oncomine.org) 25. The threshold was set as follows: *p*-value = 0.0001, fold change = 2, gene rank = 10%, data type: mRNA. Differences of *CA4* mRNA expression in different cancers and adjacent normal tissues were analyzed by Student's t test (**p < 0.01, ***p < 0.001).

### Patients and transcriptional expression profile

Available RNA-sequence data of a total of 533 KIRC patients, 530 brain lower grade glioma (LGG) patients, 517 lung adenocarcinoma (LUAD) patients, 498 prostate adenocarcinoma (PRAD) patients and 80 uveal melanoma (UVM) patients were consecutively acquired from the Cancer Genome Atlas (TCGA) database 26. Illumina HiSeq 2000 RNA Sequencing platform was utilized to experimentally measure the gene expression profiles by University of North Carolina TCGA genome characterization center. ESTIMATE algorithm was utilized for visualizing stromal and immune scores by "estimate" R package (http://r-forge.r-project.org; dependencies=TRUE, repos=rforge). Participants in each kind of tumors were divided into two groups by X-tile software, which figured out the cut-off value of* CA4* mRNA expression 27.

### Statistical analysis

Survival analyses between high and low *CA4* mRNA expression groups were performed in KIRC, LGG, LUAD, PRAD and UVM patients. Progression-free survival (PFS), the primary endpoint for patients, is the duration between the first treatment and the date of progression or death or second-line treatment. Overall survival (OS), the secondary endpoint, is the duration between first diagnosis or treatment and the last follow-up or death. Log-rank test in separate curves and Kaplan-Meier method with 95% confidence intervals (95%CI) were utilized to performing the follow-up duration analysis. To further find significant independent variables of these cancers, univariate and multivariate Cox regression analyses were performed. Phenotype and expression profiles of *CA4* among KIRC, LGG, LUAD and UVM patients were downloaded and illustrated from TCGA database. All analyses were performed in the R (Version 3.6.0), RStudio (Version 1.2.1335), IBM SPSS Statistics 25 and GraphPad Prism 8. Two-sided and *p*-values less than 0.05 were taken as significant in all tests.

### Protein-protein interaction (PPI) network construction

PPI network of *CA4* and co-expression genes was constructed in three methods in this study. First, GeneMANIA (http://www.genemania.org) was utilized for generating hypotheses about gene functions 28. Consequently, GeneMANIA was used to find functionally similar genes with *CA4* and institute a gene-gene interaction network for them on the basis of physical interactions, co-expression, predicted, co-localization, pathway, genetic interactions and shared protein domains in this study. Second, Search Tool for the Retrieval of Interacting Genes (STRING; http://string-db.org) (version 11.0) is utilized to predict PPI network between *CA4* and its co-expression genes and visualizes the functional interactions among them 29. The combined scores of the interactions greater than 0.4 were considered statistically significant. Third, PPI network of *CA4* and co-expression genes were also constructed in terms of lncRNA and PPI by R software (version 3.3.2).

### Functional annotations and molecular penal analysis

Cytoscape (version 3.6.1) is an bioinformatics software platform, which is utilized to illustrate molecular interaction network 30. ClueGO (version 2.5.4) and CluePedia (version 1.5.4) are Cytoscape plug-ins used to visualize the non-redundant biological terminology for gene modules in functional grouping networks 31,32. Gene ontology (GO), including biological process (BP), cellular component (CC) and molecular function (MF), and Kyoto Encyclopedia of Genes and Genomes (KEGG) pathways analyses for *CA4* and its co-expression genes identified in STRING were illustrated and visualized by ClueGO and CluePedia. Transcription factor regulation network was predicted using R software. Significant nodes were colored in red in line with *CA4*.

### Tumor immune interactions analysis

Tumor-immune system interactions (TISIDB; http://cis.hku.hk/TISIDB) is an repository portal to integrate multiple resources for immunological results obtained according to seven public databases 33. Interactions between immunologic system and tumor among twenty-eight tumor infiltrating lymphocytes (TILs) in 30 kinds of human cancers were investigated in this study. The associations between *CA4* expression and OS across human cancers were calculated by TISIDB using log rank test (-log10(*p*-value)).

We further calculated the relations between immune score and *CA4* mRNA expression in patients with different cancers. Scatter plots were calculated using Pearson's correlation and statistical significance. The result with the criteria (*p*-value < 0.05 and |Pearson's r| > 0.2) was considered to have significant correlation between *CA4* expression and immune score in each kind of cancers.

### Correlations of CA4 and immune cell signatures infiltrations

Tumor Immune Estimation Resource (TIMER, https://cistrome.shinyapps.io/timer/) database contains 10,897 subjects among thirty-two cancers from TCGA for estimating various immune infiltrations 34. TIMER is utilized to analyze relationships between *CA4* expression and immune infiltration levels. Spearman's correlation was statistically calculated to generate scatter plots. Moreover, correlation analysis was used to illustrate relationships between *CA4* and gene markers of diverse tumor-infiltrating immune cells. The result with the criteria (*p*-value < 0.05 and |Pearson's r| > 0.2) was considered to have significant correlation between them.

Correlation analysis between *CA4* and gene markers of immune cells in both TIMER and Gene Expression Profiling Interactive Analysis (GEPIA) (http://gepia.cancer-pku.cn/index.html). Spearman's correlation analysis was performed for *CA4* and gene markers of diverse tumor-infiltrating immune cells. The result with the criteria (*p*-value < 0.05 and |Pearson's r| > 0.4) was considered to have significant correlation between them.

### CA4 expression in KIRC and normal samples

CA4 protein expression, coded by *CA4* gene, was detected in KIRC and normal samples from the human protein atlas (https://www.proteinatlas.org/) and immunohistochemistry (IHC) data, including staining quantity, intensity, location and patients' data was available online. Formalin-fixed, paraffin-embedded KIRC tissues and human renal tissues were stained for anti-CA4 using ab236315 (Abcam, USA) at 1/3000 dilution in Fudan University Shanghai Cancer Center (FUSCC) cohort, and then independently evaluated by two experienced pathologists. The overall IHC score ranging from 0 to 12 was measured based on the multiply of the staining intensity and extent score, as previously described. Low CA4 expression group scores from 0 to 2, and high CA4 group scores from 3 to 12.

## Results

This study is composed of three stages. First and foremost, we compared mRNA expression of *CA4* between different tumors and corresponding normal tissues according to datasets hosted on TCGA platforms; then, survival analysis was performed using TISIDB platform and TCGA cohort on the basis of distinct comparison expression of *CA4* in five kinds of tumors; finally, molecular penal analysis and functional annotations of *CA4*-related genes was elaborated and correlations between *CA4* mRNA expression and TIME were analyzed in detail.

### Expression levels of CA4 in various human cancers and paired normal tissues

**Figure 1:**
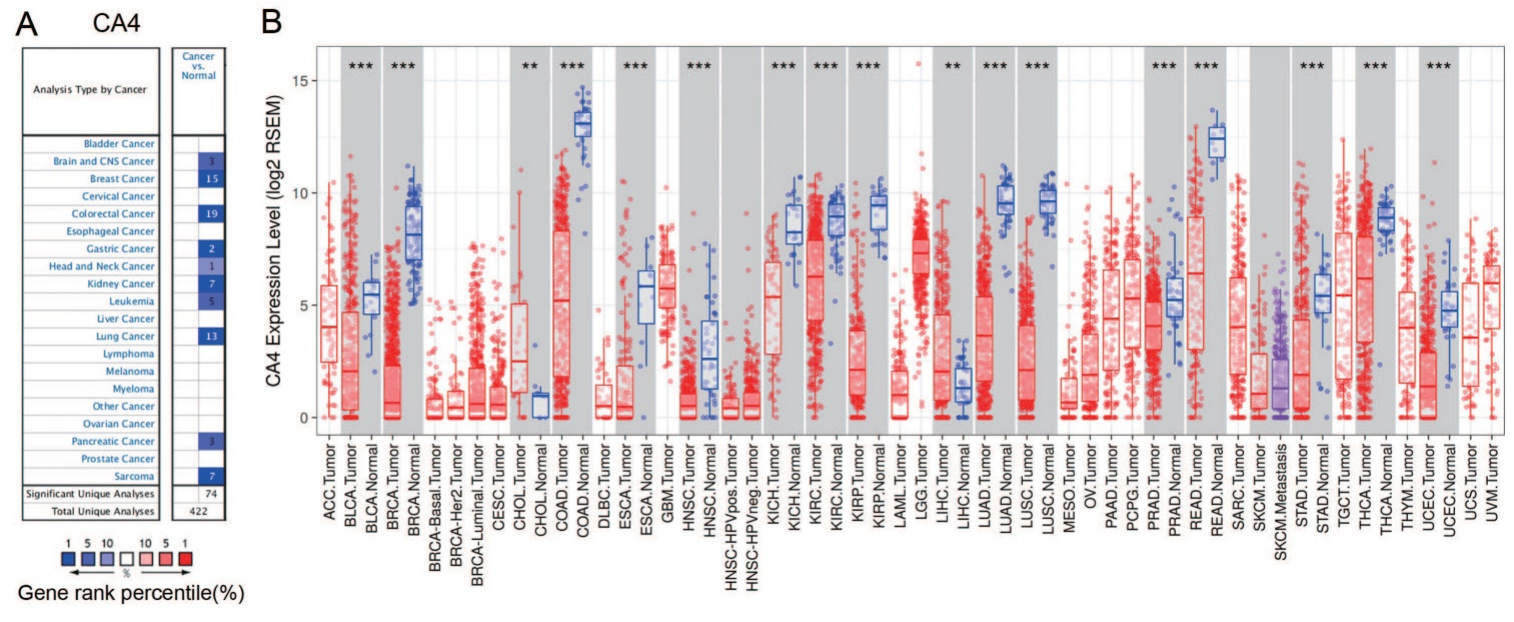
***CA4* expression levels in different types of human cancers and paired normal tissues.** (A) Decreased *CA4* in datasets of different types of cancers compared with normal tissues from Oncomine database. (B) Relative *CA4* mRNA expression levels in different cancers and adjacent normal tissues were determined from TCGA database using Student's t test (**p < 0.01, ***p < 0.001). TCGA, the cancer genome atlas.

The differential mRNA expression of *CA4* in various human cancers and paired normal tissues were compared on the basis of datasets released from the Oncomine and TIMER platform. Decreased *CA4* mRNA expressions were found in datasets of brain and CNS cancer, breast cancer, colorectal cancer, gastric cancer, head and neck cancer, kidney cancer, leukemia cancer, lung cancer, pancreatic cancer and sarcoma compared to normal tissues from Oncomine database (**Figure 1A**). Moreover, relative *CA4* mRNA expression levels between different cancers and corresponding normal tissues were determined based on TCGA database using Student's t test (**Figure 1B**). *CA4* mRNA expressions were found significantly lower in bladder urothelial carcinoma (BLCA), breast invasive carcinoma (BRCA), colon adenocarcinoma (COAD), esophageal carcinoma (ESCA), head and neck squamous cell carcinoma (HNSC), kidney chromophobe (KICH), KIRC, kidney renal papillary cell carcinoma (KIRP), LUAD, lung squamous cell carcinoma (LUSC), PRAD, rectum adenocarcinoma (READ), stomach adenocarcinoma (STAD), thyroid carcinoma (THCA) and uterine corpus endometrial carcinoma (UCEC) compared to the corresponding normal tissues. Nevertheless, *CA4* mRNA expressions were found significantly higher in cholangiocarcinoma (CHOL) and liver hepatocellular carcinoma (LIHC) compared to the corresponding normal tissues (**p < 0.01, ***p < 0.001).

### Prognostic value of CA4 in different cancers and Cox regression analyses of TCGA cohorts

**Figure 2:**
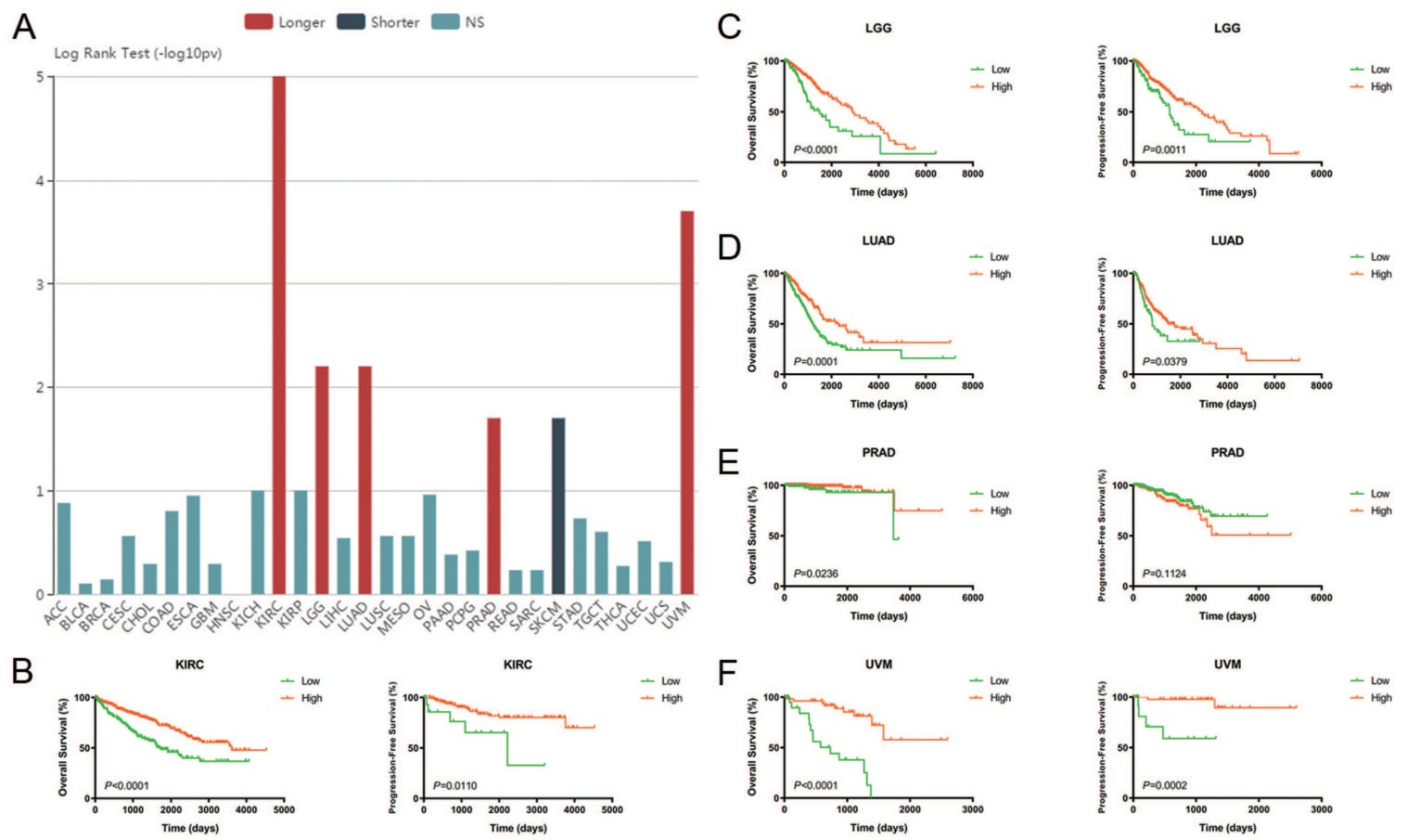
** Prognostic value of *CA4* in different cancers and its significant survival benefits in KIRC, LGG, LUAD, PRAD and UVM.** (A) Prognostic implication of *CA4* in 30 different tumors from TCGA database using log rank test (-log10(p-value)). (B-F) Survival analysis of *CA4* was performed in KIRC, LGG, LUAD, PRAD and UVM from TCGA database using Kaplan-Meier methods. Decreased expression of *CA4* significantly correlated with worse OS and PFS in KIRC, LGG, LUAD and UVM, and worse OS of PRAD. KIRC, kidney renal clear cell carcinoma; LGG, brain lower grade glioma; LUAD, lung adenocarcinoma; PRAD, prostate adenocarcinoma; UVM, uveal melanoma; TCGA, the cancer genome atlas; OS, overall survival; PFS, progression-free survival.

The relationships between *CA4* mRNA expression and prognosis of cancer patients were investigated. Prognostic implication of *CA4* in 30 different tumors from TCGA database using log rank test (-log10(*p*-value)) (**Figure 2A**). Notably, increased expression of *CA4* significantly correlated with better OS in KIRC, LGG, LUAD, PRAD and UVM, while worse OS in SKCM. Survival analysis of CA4 was further performed in KIRC, LGG, LUAD, PRAD and UVM from TCGA database using Kaplan-Meier methods. Decreased expression of *CA4* was significantly related to worse OS and PFS in KIRC, LGG, LUAD and UVM, and worse OS of PRAD (*p* < 0.05) (**Figure 2B-F**).

Subsequently, Cox regression analyses were performed in KIRC, LGG, LUAD and UVM in TCGA cohorts. Depending on univariate Cox regression analysis models, pT stage, pM stage, AJCC stage and ISUP grade significantly correlated to both PFS and OS in KIRC patients. Gender was significantly relevant to PFS while age and pN stage significantly correlated to OS in KIRC patients (*p* < 0.05; **Supplementary Figure 1A-B**). Depending on univariate Cox regression analysis models, age and neoplasm grade significantly correlated to both PFS and OS and histological type significantly correlated to OS in LGG patients (*p* < 0.05; **Supplementary Figure 1C-D**). Depending on univariate Cox regression analysis models, pT stage, pN stage and AJCC stage significantly correlated to both PFS and OS and pM stage significantly correlated to OS in LUAD patients (*p* < 0.05; **Supplementary Figure 1E-F**). Depending on univariate Cox regression analysis models, cell type was significantly relevant to both PFS and OS in UVM patients. Meanwhile, age and tumor basal diameter significantly correlated to OS in UVM patients (*p* < 0.05; **Supplementary Figure 1G-H**). Remarkably, *CA4* amplification was obviously related to better PFS (KIRC: hazard ratio [HR] = 0.661, *p* < 0.001; LGG: HR = 0.552, *p* = 0.002; LUAD: HR = 0.922, *p* = 0.020; UVM: HR = 0.454, *p* = 0.001) and better OS (KIRC: HR = 0.847, *p* < 0.001; LGG: HR = 0.552, *p* < 0.001; LUAD: HR = 0.918, *p* = 0.007; UVM: HR = 0.454, *p* < 0.001) in all of these four cancers.

**Figure 3:**
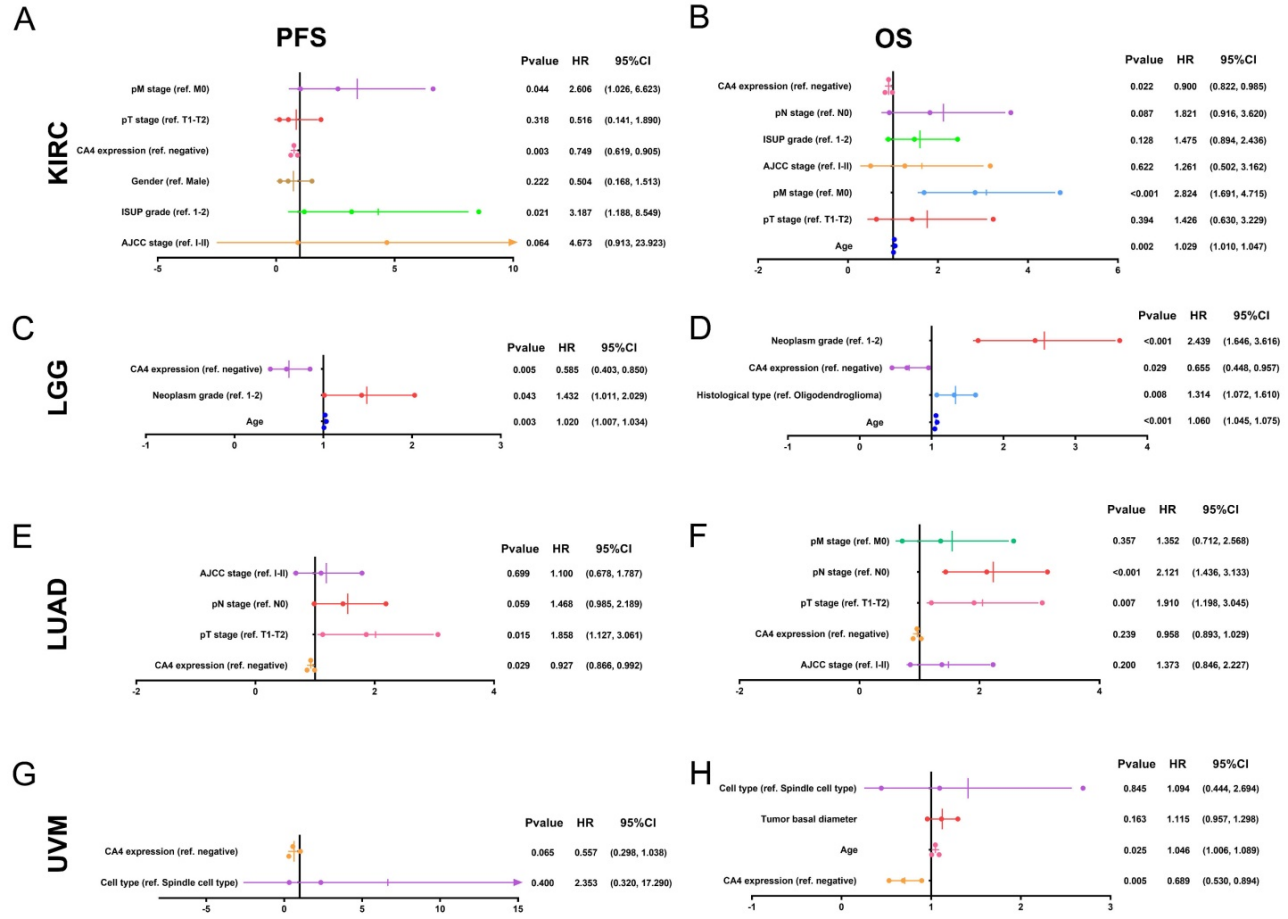
** Multivariate Cox regression analysis of PFS and OS of *CA4* in KIRC, LGG, LUAD and UVM patients from TCGA cohort.** (A-B) pM stage was significantly relevant to both PFS and OS in KIRC patients. Meanwhile, ISUP grade and age were significantly relevant to PFS or OS in KIRC patients, respectively (*p* < 0.05). (C-D) Age and neoplasm grade were significantly relevant to both PFS and OS in LGG patients. Meanwhile, histological type significantly correlated to OS in LGG patients (*p* < 0.05). (E-F) pT stage was significantly relevant to both PFS and OS and pN stage was significantly relevant to OS in LUAD patients (*p* < 0.05). (G-H) Age was significantly relevant to OS in UVM patients (*p* < 0.05). Notably, *CA4* amplification obviously correlated with better PFS in KIRC, LGG and LUAD (KIRC: HR = 0.749, *p* = 0.003; LGG: HR = 0.585, *p* = 0.005; LUAD: HR = 0.927, *p* = 0.029) and better OS in KIRC, LGG and UVM (KIRC: HR = 0.900, *p* = 0.022; LGG: HR = 0.655, *p* = 0.029; UVM: HR = 0.689, *p* = 0.005). PFS, progression-free survival; OS, overall survival; KIRC, kidney renal clear cell carcinoma; LGG, brain lower grade glioma; LUAD, lung adenocarcinoma; UVM, uveal melanoma; TCGA, the cancer genome atlas.

In multivariate Cox regression analysis models, pM stage was still significantly relevant to both PFS and OS in KIRC patients. Meanwhile, ISUP grade and age were significantly relevant to PFS or OS in KIRC patients, respectively (*p* < 0.05; **Figure 3A-B**). In multivariate Cox regression models, age and neoplasm grade were still significantly relevant to both PFS and OS in LGG patients. Meanwhile, histological type significantly correlated to OS in LGG patients (*p* < 0.05; **Figure 3C-D**). In multivariate Cox regression models, pT stage was still significantly relevant to both PFS and OS and pN stage was significantly relevant to OS in LUAD patients (*p* < 0.05; **Figure 3E-F**). In multivariate Cox regression models, age was still significantly relevant to OS in UVM patients (*p* < 0.05; **Figure 3G-H**). Notably, *CA4* amplification obviously correlated with better PFS in KIRC, LGG and LUAD (KIRC: HR = 0.749, *p* = 0.003; LGG: HR = 0.585, *p* = 0.005; LUAD: HR = 0.927, *p* = 0.029) and better OS in KIRC, LGG and UVM (KIRC: HR = 0.900, *p* = 0.022; LGG: HR = 0.655, *p* = 0.029; UVM: HR = 0.689, *p* = 0.005).

### Molecular panel analysis and functional annotations of CA4-related genes

**Figure 4:**
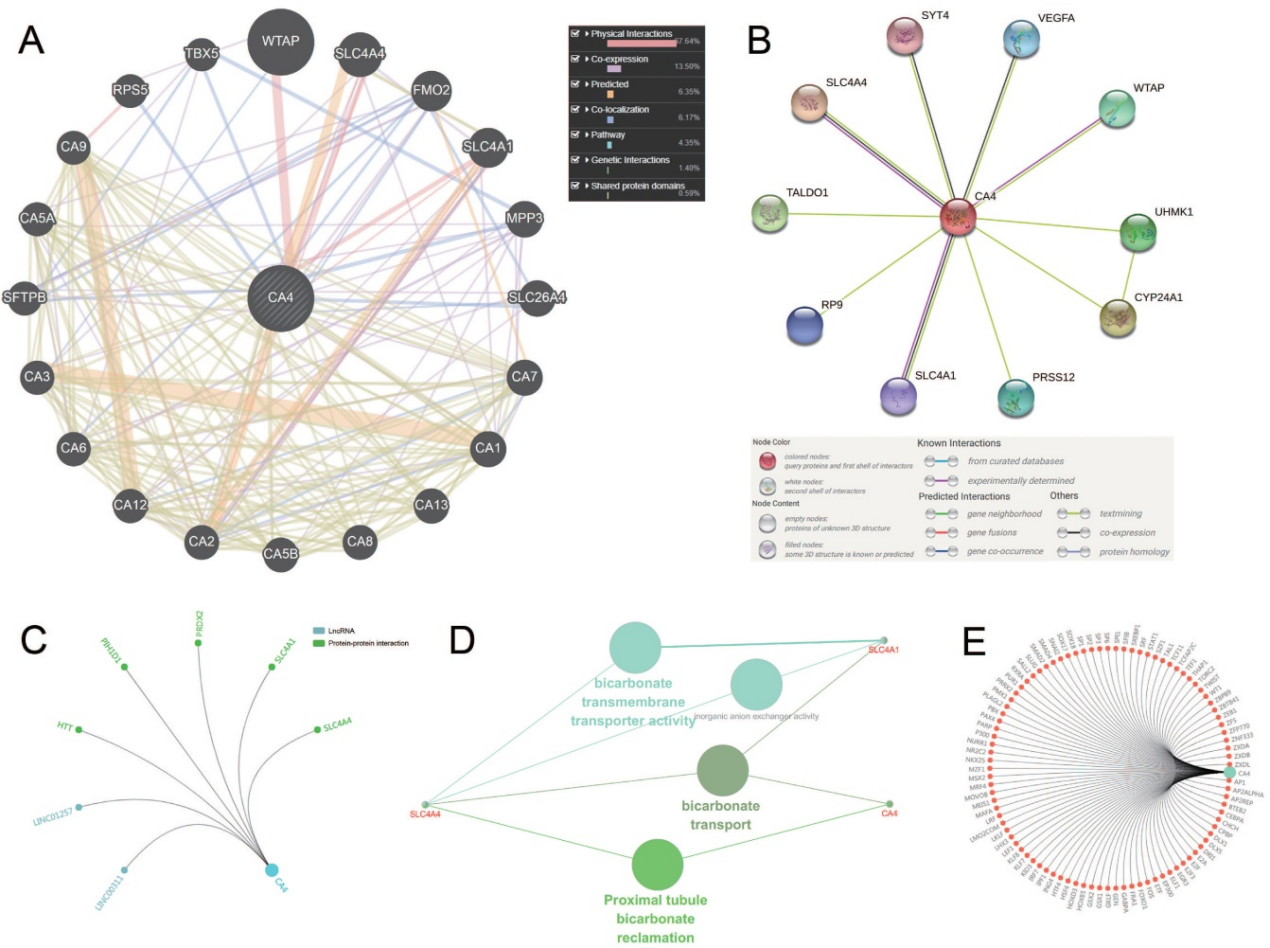
** Molecular penal ananlysis and functional annotations of *CA4*-related genes.** (A) Gene-gene interaction of *CA4* and related genes. The size of the nodes represents the strength of interactions. Different line colors represent different types of gene-gene interactions. (B) PPI network of *CA4* and its co-expression genes was constructed visually. Different line colors represent different types of protein-protein interactions. (C) PPI network was constructed in *CA4*. Significant nodes were marked in different colors in line with *CA4* (LncRNA and PPI). (D) Functional annotations indicated the changes in biological processes of *CA4* significantly correlated with bicarbonate transmembrane transporter activity, bicarbonate transport and proximal tubule bicarbonate reclamation using ClueGO. (E) Transcription factor network was predicted. Significant nodes were marked in red in line with *CA4*. PPI, Protein-protein interaction.

We use three methods to identify the co-expression network of *CA4*. Gene-gene interaction of *CA4* and related genes was constructed using GeneMANIA database (**Figure 4A**). CA4 was surrounded by 20 nodes which represented closely related genes in terms of physical interactions (67.64%), co-expression (13.50%), predicted (6.35%), co-localization (6.17%), pathway (4.35%), genetic interactions (1.40%) and shared protein domains (0.59%). The size of the nodes represents the strength of interactions. Different line colors represent different types of gene-gene interactions. In addition, PPI network between *CA4* and co-expression genes was illustrated using STRING (**Figure 4B**). Different line colors represent different types of protein-protein interactions. Meanwhile, PPI network was constructed in *CA4* using R software. Then mark significant nodes with diverse colors in line with *CA4* (LncRNA and PPI) (**Figure 4C**). Importantly, Solute carrier family 4 member 1 (*SLC4A1*) and Solute carrier family 4 member 4 (*SLC4A4*) were identified by these three methods. WT1 associated protein (*WTAP*) was identified by both GeneMINIA and STRING. Functional annotations indicated the changes in biological processes of *CA4* significantly correlated with bicarbonate transmembrane transporter activity, bicarbonate transport and proximal tubule bicarbonate reclamation using ClueGO (**Figure 4D**). Transcription factor network was predicted in **Figure 4E**. Significant nodes were marked in red in line with *CA4*.

### Role of CA4 in different TIMEs

**Figure 5:**
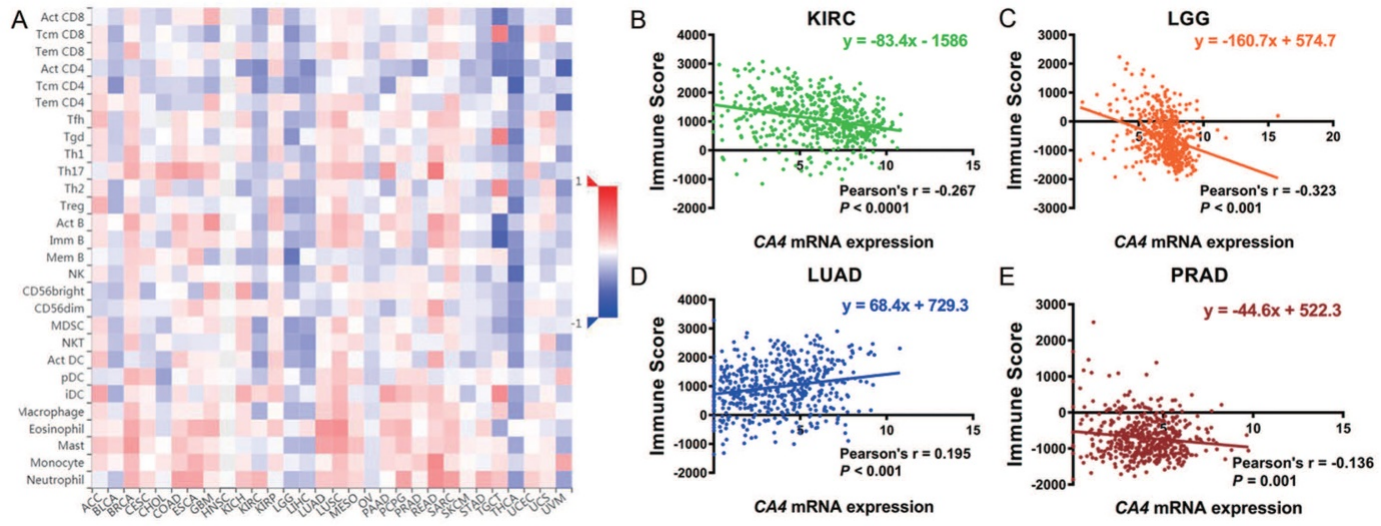
** Role of *CA4* in different tumor immune microenvironments.** (A) Relations between *CA4* expression and the abundance of TILs in different tumors. (B-E) Relations between immune score and *CA4* mRNA expression in KIRC, LGG, LUAD and PRAD were performed. Scatter plots were calculated using Pearson's correlation and statistical significance. *CA4* was obviously correlated with immune score in KIRC and LGG (|Pearson's r| > 0.2). TILs, tumor infiltrating lymphocytes; KIRC, kidney renal clear cell carcinoma; LGG, brain lower grade glioma; LUAD, lung adenocarcinoma; PRAD, prostate adenocarcinoma.

**Figure 6:**
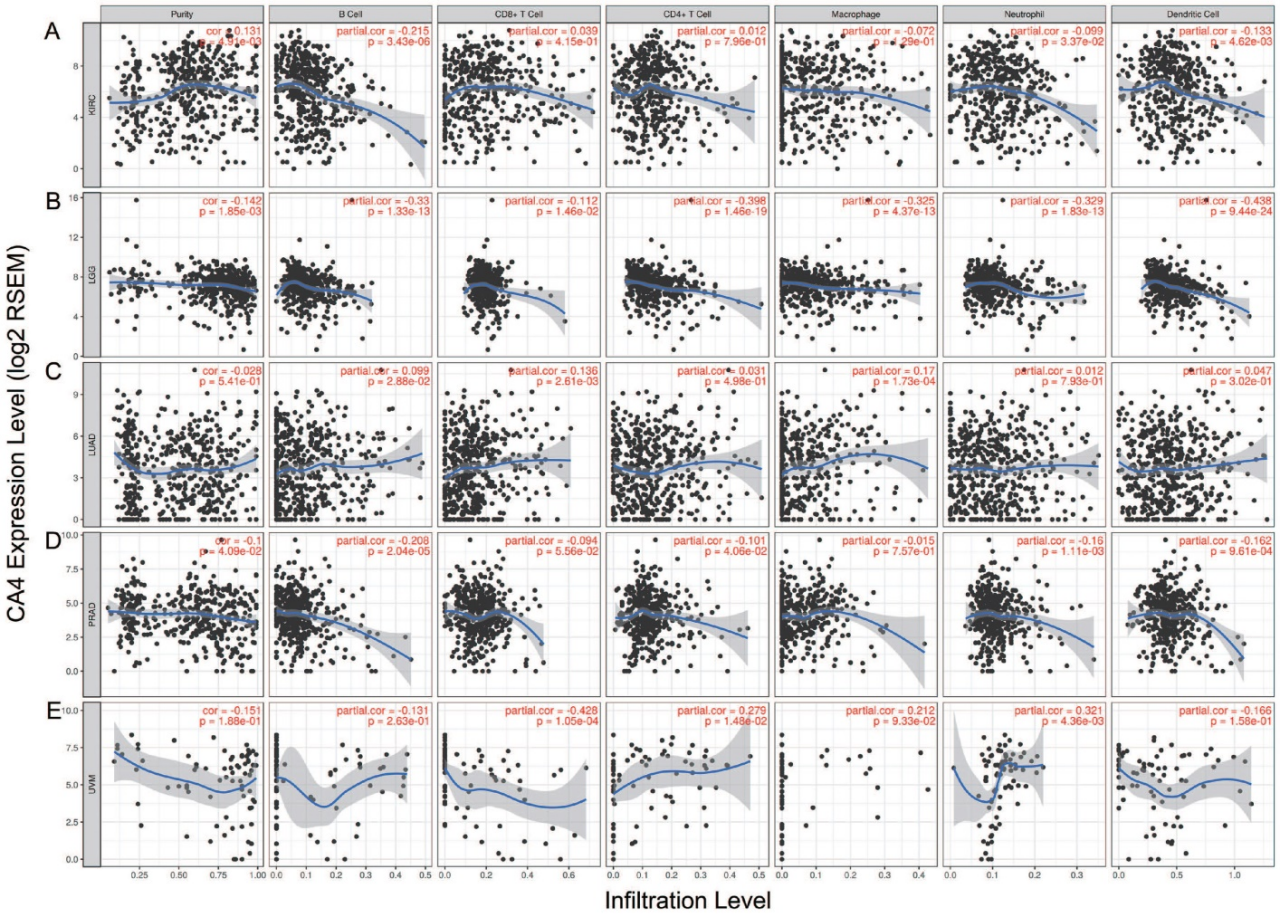
** Immune infiltration of *CA4* in five cancers using TIMER.** Correlation analysis between *CA4* and immune infiltration levels in KIRC (A), LGG (B), LUAD (C), PRAD (D) and UVM (E) was performed. Spearman's correlation and statistical significance were performed to generate scatter plots. TIMER, Tumor Immune Estimation Resource; KIRC, kidney renal clear cell carcinoma; LGG, brain lower grade glioma; LUAD, lung adenocarcinoma; PRAD, prostate adenocarcinoma; UVM, uveal melanoma.

We further explored the different TIMEs of *CA4* in 30 kinds of tumors and calculated the immune scores correlated with *CA4* in KIRC, LGG, LUAD and PRAD. Relations between *CA4* expression and the abundance of TILs in different tumors were performed in heat map (**Figure 5A**). Additionally, relations between immune scores and *CA4* mRNA expression in KIRC, LGG, LUAD and PRAD were performed (**Figure 5B-E**). Scatter plots were calculated using Pearson's correlation and statistical significance. *CA4* was obviously correlated with immune score in KIRC and LGG (|Pearson's r| > 0.2). Immune infiltration levels of *CA4* in KIRC, LGG, LUAD, PRAD and UVM were performed using TIMER, respectively (**Figure 6**). Partial Spearman's correlation and statistical significance were calculated for generating scatter plots. *CA4* expression levels significantly correlated to B cell infiltration in KIRC, B cell, CD4^+^ T cell, macrophage, neutrophil and dendritic cell infiltration in LGG, B cell infiltration in PRAD, CD8^+^ T cell, CD4^+^ T cell and neutrophil infiltration in UVM (|partial.cor| > 0.2 and *p*<0.05). Spearman's correlation and estimated statistical significance between *CA4* expression and related genes and markers of immune cells were displayed among KIRC, LGG, LUAD and UVM using TIMER (**Table 1**) and GEPIA (**Table 2**), respectively. Important markers of various immune cells were illustrated at great length, including CD8^+^ T cell, T cell (general), B cell, monocyte, tumor-associated macrophage (TAM), M1 macrophage, M2 macrophage, neutrophils, natural killer cell, dendritic cell, T helper cell 1, T helper cell 2, Follicular helper T cell, T helper cell 17, regulatory T cell, T cell exhaustion (*p < 0.05, **p < 0.01, ***p < 0.001, ****p < 0.0001).

### Differential CA4 expression in KIRC

**Figure 7:**
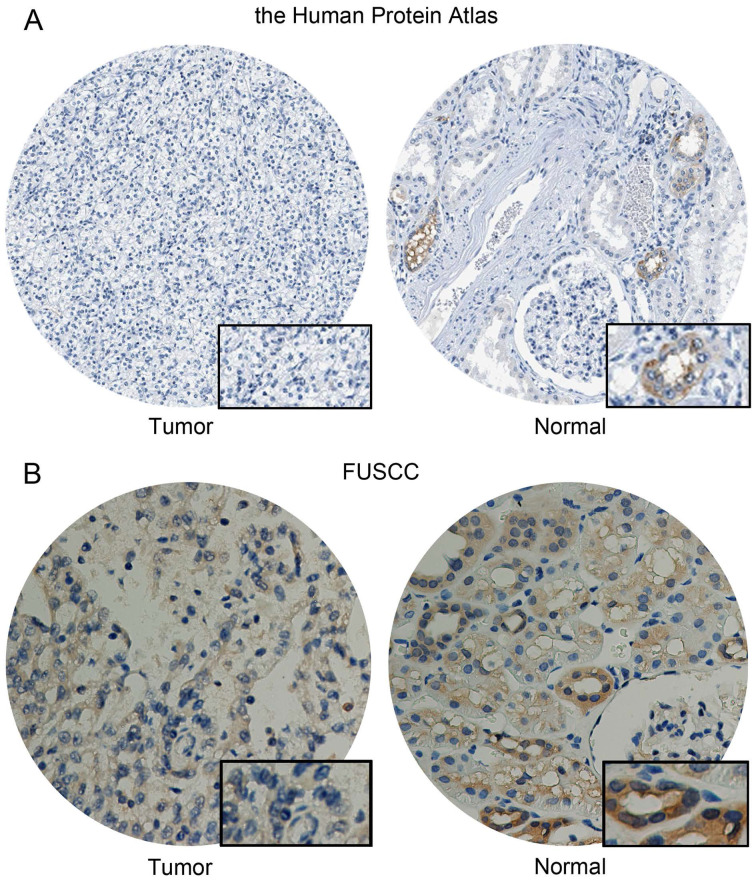
** Differential CA4 expression in 322 KIRC patients from FUSCC cohorts.** (A) CA4 was detected low expressed in normal cells of kidney tubules, while not detected in KIRC tissues from the Human Protein atlas. (B) Significantly elevated CA4 expression was found in normal tissues compared with KIRC tissues from FUSCC cohort. KIRC, kidney renal clear cell carcinoma; FUSCC, Fudan University Shanghai Cancer Center.

CA4 was detected low expressed in normal cells of kidney tubules, while not detected in KIRC tissues from the Human Protein atlas (**Figure 7A**). Meanwhile, significantly elevated CA4 expression was found in normal tissues compared with KIRC tissues from FUSCC cohort (**Figure 7B**).

## Discussion

CA isoenzymes make biochemical reaction with other enzymes in diverse ways. CA isoenzymes are triggered up- or down-regulated on the level of genes by normal pathways changes in diverse dysfunctions, which contributes to the major prerequisite of CA as a biomarker 11. Of all Carbonic Anhydrase family, CA2 is the most active CA isoenzyme. The hydration rate of CO_2_ is close to the diffusion limit, and it has most wide distribution in human. It is expressed in the cytoplasm of almost every tissue or organ 11. Parkilla and his collaborators lately suggested *CA2* acted as a marker of gastrointestinal stromal tumors. Compared with low or no protein expression, high expression of *CA2* is related to better survival outcomes, suggesting that *CA2* can be a potential marker for this interstitial tumor diagnosis 35.

CA4, a fast isoenzyme similar to CA2, binds to the membrane through glycosylphosphatidylinositol anchors 11. It was found that CA4 resisted to the restraint of halogen ions better than CA2 and was suitable for catalyzing the interconversion of CO_2_/HCO_3_^-^. *CA4* was reported to express on certain capillary beds, the parietal membrane of kidney, pulmonary microvessels and choroidal capillaries 36.

In our study, *CA4* mRNA expressions were found significantly lower in 15 kinds of cancers compared to corresponding normal tissues. However, *CA4* expression was found higher within CHOL and LIHC compared to the corresponding normal tissues. Markedly, the decreased *CA4* expression is closely related to worse OS and PFS in KIRC, LGG, LUAD and UVM, as well as worse OS within PRAD (p <0.05). This evidence suggests that *CA4* plays an anti-tumor role in the four tumors previously described. Therefore, understanding of the mechanism of *CA4* promoting tumorigenesis and progression may provide strategies for clinical treatment of tumors.

Tumor microenvironment plays a key part in tumor generating, development, aggression and metastasis 2. Invasive tumors are usually described into various cancer types, along with diverse cells associated with innate and adaptive immunity 37. Lymphocyte is usually considered as the most important tumor-infiltrating immune cell, such as T cell, B cell and NK cell. TILs were often existed in cancers and were considered to be the host's immune response to malignant cells, which reflected actional approaches of “cancer immunoediting” 38. Over the past few decades, increasing studies suggested that better prognosis for many cancers correlated with TILs, indicating the valuable function in resisting tumor development 37,39. TIL represents a heterogeneous cell population such as the immune subgroups categorized depending on the effects of physiology and pathology in the context of immunity. Accordingly, tumor microenvironment may be composed of sophisticated TILs with the consequence of competitive immunostimulatory or immunosuppressive effects. Therefore, TILs is important in regulating anti-tumor immune system 40. The close relationship between clinical results and corresponding TILs was confirmed among diverse cancers, such as melanoma 41 and lung cancer 42. In colon cancer, TILs are considered to be better predictors for prognosis, even compared with classic prognostic factors such as TNM staging 43. For this reason, it was suggested that “Immunoscore”, an designated scoring system, was on the basis of 3 immune components: type, density and location of immune cells 37.

Nowadays, TILs also attract more attentions for cancer immunotherapy because they may be used to be markers for identifying patients who are possibly suitable for immunosuppressive therapy. Immunotherapy as a treatment for cancer has sparked new interest because of hopeful clinical outcomes found in the use of inhibiting immune checkpoint within a variety of cancers, including Hodgkin's disease and melanoma 44,45. In this study, we explored different TIMEs of *CA4* in 30 kinds of tumors and calculated the immune scores correlated with *CA4* in KIRC, LGG, LUAD and PRAD. Relations between *CA4* expression and the abundance of TILs in different tumors were performed in heat map. Additionally, relations between immune scores and *CA4* mRNA expression in KIRC, LGG, LUAD and PRAD were performed. These results showed that increased *CA4* expression correlated with better prognosis and *CA4* expression levels significantly correlated with B cell infiltration within KIRC, B cell, CD4^+^ T cell, macrophage, neutrophil and dendritic cell infiltration within LGG, B cell infiltration in PRAD, CD8^+^ T cell, CD4^+^ T cell and neutrophil infiltration within UVM (|partial.cor| > 0.2 and *p*<0.05). Therefore, *CA4* may play a crucial part in immune cell infiltration and prognosis.

Nevertheless, several limitations should be considered in this study. Most parts of this study were performed in silico and were investigated based on large-scale samples from TCGA. The lack of independent cohorts of patients to validate the prognostic values of *CA4* in LGG, LUAD, PRAD and UVM need to be considered. However, considering we have validated significant prognostic implications of *CA4* in KIRC, more investigative researches should be performed to further elucidate *CA4* as potential biomarker for diagnosis, immunotherapy and prognosis in these cancers in the future. The specific molecular functions of *CA4* mRNA expression also need further researches to clarify.

## Conclusion

Consequently, our data revealed that decreased *CA4* expression was related to worse prognosis in multiple cancers, especially in KIRC, LGG, LUAD, PRAD and UVM. In addition, *CA4* is possible to play an important part in immune cell infiltration among these five cancers. Therefore, CA4 is suggested to provide a novel biomarker for diagnosis, immunotherapy and prognosis in these cancers.

## Supplementary Material

Supplementary figure.

## Figures and Tables

**Table 1:** Correlation analysis between CA4 and related genes and markers of immune cells in TIMER.

Description	Gene markers	KIRC						LGG						LUAD						UVM				
		None			Purity			None			Purity			None			Purity			None			Purity	
		Cor	P		Cor	P		Cor	P		Cor	P		Cor	P		Cor	P		Cor	P		Cor	P
CD8+ T cell	CD8A	-0.253	****		-0.249	****		0.149	***		0.116	*		0.094	*		0.094	*		-0.303	**		-0.256	*
	CD8B	-0.201	****		-0.191	****		0.175	****		0.131	**		0.06	0.171		0.059	0.195		-0.33	**		-0.277	*
T cell (general)	CD3D	-0.258	****		-0.241	****		-0.178	****		-0.218	****		0.054	0.222		0.038	0.397		-0.34	**		-0.298	**
	CD3E	-0.261	****		-0.242	****		-0.19	****		-0.22	****		0.102	*		0.104	*		-0.238	*		-0.192	0.0935
	CD2	-0.274	****		-0.256	****		-0.212	****		-0.239	****		0.105	*		0.105	*		-0.321	**		-0.275	*
B cell	CD19	-0.27	****		-0.243	****		-0.125	**		-0.13	**		0.073	0.0983		0.056	0.214		-0.038	0.738		-0.017	0.88
	CD79A	-0.267	****		-0.262	****		-0.269	****		-0.308	****		0.001	0.89		-0.024	0.6		0.075	0.506		0.081	0.486
Monocyte	CD86	-0.338	****		-0.34	****		-0.316	****		-0.395	****		0.017	0.703		0.003	0.942		-0.34	**		-0.308	**
	CD115 (CSF1R)	-0.241	****		-0.224	****		-0.264	****		-0.353	****		0.036	0.414		0.031	0.491		-0.135	0.232		-0.126	0.274
TAM	CCL2	0.218	****		0.256	****		-0.3	****		-0.327	****		-0.063	0.156		-0.084	0.0617		0.187	0.095		0.22	0.0548
	CD68	-0.339	****		-0.367	****		-0.355	****		**-0.412**	****		0.153	***		0.152	***		0.16	0.157		0.215	0.0603
	IL10	-0.215	****		-0.197	****		-0.364	****		**-0.4**	****		0.151	***		0.147	**		-0.136	0.229		-0.104	0.368
M1 Macrophage	INOS (NOS2)	0.354	****		0.374	****		0.257	****		0.241	****		0.267	****		0.275	****		0.098	0.388		0.143	0.215
	IRF5	-0.298	****		-0.305	****		-0.25	****		-0.332	****		0.036	0.41		0.027	0.547		-0.3	**		-0.249	*
	COX2 (PTGS2)	-0.051	0.24		0.005	0.923		0.032	0.469		0.007	0.871		-0.112	*		-0.101	*		**-0.445**	****		**-0.426**	***
M2 Macrophage	CD163	-0.18	****		-0.19	****		-0.398	****		-0.398	****		0.138	**		0.141	**		-0.213	0.0581		-0.19	0.098
	VSIG4	-0.262	****		-0.262	****		-0.366	****		**-0.418**	****		0.145	***		0.135	**		-0.215	0.0557		-0.198	0.0842
	MS4A4A	-0.233	****		-0.223	****		-0.347	****		-0.371	****		0.155	***		0.154	***		-0.151	0.182		-0.121	0.296
Neutrophils	CD66b (CEACAM8)	0.093	*		0.095	*		-0.056	0.204		-0.045	0.332		0.212	****		0.209	****		NA	NA		NA	NA
	CD11b (ITGAM)	-0.273	****		-0.271	****		-0.282	****		-0.373	****		0.039	0.374		0.026	0.558		0.057	0.617		0.063	0.585
	CCR7	-0.208	****		-0.199	****		-0.134	**		-0.16	***		0.182	****		0.192	****		-0.095	0.401		-0.064	0.58
Natural killer cell	KIR2DL1	0.195	****		0.176	***		-0.058	0.187		-0.05	0.273		0.178	****		0.182	****		-0.141	0.212		-0.151	0.189
	KIR2DL3	0.106	*		0.087	0.0627		-0.104	*		-0.113	*		0.122	**		0.106	*		-0.359	**		-0.329	**
	KIR2DL4	-0.039	0.369		-0.034	0.461		-0.304	****		-0.329	****		-0.074	0.095		-0.076	0.0929		-0.211	0.0598		-0.168	0.143
	KIR3DL1	0.238	****		0.202	****		-0.018	0.676		-0.013	0.774		0.189	****		0.194	****		-0.147	0.192		-0.111	0.335
	KIR3DL2	0.012	0.789		-0.011	0.814		-0.087	*		-0.093	*		0.021	0.639		0.014	0.757		-0.368	***		-0.329	**
	KIR3DL3	-0.029	0.511		-0.008	0.864		-0.057	0.196		-0.045	0.322		-0.024	0.58		-0.021	0.644		-0.257	*		-0.229	*
	KIR2DS4	0.065	0.136		0.06	0.195		-0.088	*		-0.109	*		0.081	0.0656		0.078	0.083		-0.28	*		-0.25	*
Dendritic cell	HLA-DPB1	-0.159	***		-0.158	***		-0.298	****		-0.332	****		0.17	***		0.178	****		-0.142	0.208		-0.11	0.34
	HLA-DQB1	-0.049	0.258		-0.041	0.375		-0.244	****		-0.271	****		0.14	**		0.126	**		-0.254	*		-0.218	0.0567
	HLA-DRA	-0.192	****		-0.209	****		-0.348	****		-0.378	****		0.125	**		0.121	**		-0.254	*		-0.263	*
	HLA-DPA1	-0.186	****		-0.188	****		-0.329	****		-0.363	****		0.122	**		0.126	**		-0.238	*		-0.209	0.0683
	BDCA-1 (CD1C)	0.116	**		0.156	***		-0.184	****		-0.197	****		0.204	****		0.202	****		0.106	0.349		0.139	0.229
	BDCA-4 (NRP1)	0.24	****		0.261	****		-0.208	****		-0.172	***		-0.061	0.166		-0.065	0.146		-0.299	**		-0.252	*
	CD11c (ITGAX)	-0.316	****		-0.319	****		-0.123	**		-0.172	***		0.1	*		0.097	*		-0.395	***		-0.355	**
Th1	T-bet (TBX21)	0.057	0.193		0.084	0.0706		-0.187	****		-0.173	***		0.174	****		0.174	***		-0.356	**		-0.313	**
	STAT4	-0.169	****		-0.148	**		**0.423**	****		0.397	****		0.035	0.431		0.024	0.602		-0.301	**		-0.287	*
	STAT1	-0.277	****		-0.28	****		-0.278	****		-0.267	****		-0.051	0.244		-0.052	0.249		**-0.41**	***		-0.368	***
	IFN-γ (IFNG)	-0.303	****		-0.301	****		-0.114	**		-0.141	**		0.002	0.956		-0.011	0.801		-0.362	***		-0.337	**
	TNF-α (TNF)	-0.066	0.127		-0.058	0.214		-0.133	**		-0.157	***		0.001	0.977		-0.018	0.691		-0.378	***		-0.34	**
Th2	GATA3	-0.119	**		-0.091	0.0521		-0.241	****		-0.27	****		-0.038	0.388		-0.051	0.262		-0.281	*		-0.219	0.0559
	STAT6	0.014	0.746		0.02	0.668		0.104	*		0.03	0.517		0.289	****		0.284	****		-0.07	0.539		-0.042	0.716
	STAT5A	-0.379	****		-0.372	****		-0.238	****		-0.299	****		0.09	*		0.095	*		**0.455**	****		**0.434**	****
	IL13	0.017	0.704		0.046	0.324		0.067	0.127		0.069	0.131		0.041	0.358		0.016	0.72		-0.292	**		-0.271	*
Tfh	BCL6	-0.111	*		-0.13	**		-0.262	****		-0.241	****		0.037	0.403		0.057	0.21		0.112	0.321		0.09	0.437
	IL21	-0.22	****		-0.21	****		-0.1	*		-0.094	*		0.03	0.504		0.041	0.364		-0.26	*		-0.235	*
Th17	STAT3	-0.095	*		-0.091	0.0514		**-0.519**	****		**-0.507**	****		0.049	0.262		0.066	0.143		**-0.52**	****		**-0.504**	****
	IL17A	-0.063	0.146		-0.009	0.846		-0.013	0.766		0.009	0.852		0.1	*		0.109	*		NA	NA		NA	NA
Treg	FOXP3	-0.388	****		-0.368	****		0.186	****		0.172	***		-0.071	0.106		-0.098	*		-0.243	*		-0.204	0.0745
	CCR8	-0.323	****		-0.31	****		-0.051	0.248		-0.055	0.233		-0.016	0.723		-0.028	0.532		-0.288	**		-0.241	*
	STAT5B	0.258	****		0.241	****		-0.25	****		-0.228	****		0.175	****		0.178	****		0.155	0.17		0.141	0.22
	TGFβ (TGFB1)	-0.299	****		-0.263	****		-0.296	****		-0.353	****		-0.029	0.504		-0.027	0.551		0.079	0.488		0.111	0.336
T cell exhaustion	PD-1 (PDCD1)	-0.266	****		-0.259	****		-0.27	****		-0.27	****		-0.011	0.807		-0.021	0.648		**-0.416**	***		-0.376	***
	CTLA4	-0.29	****		-0.285	****		-0.065	0.142		-0.079	0.0856		0.017	0.705		-0.002	0.962		-0.313	**		-0.273	*
	LAG3	-0.303	****		-0.282	****		-0.254	****		-0.26	****		-0.069	0.12		-0.082	0.0689		-0.391	***		-0.346	**
	TIM-3 (HAVCR2)	-0.083	0.0561		-0.103	*		-0.305	****		-0.38	****		0.047	0.284		0.035	0.444		-0.258	*		-0.229	*
	GZMB	0.008	0.849		0.034	0.467		-0.048	0.273		-0.045	0.327		-0.031	0.485		-0.047	0.303		-0.361	***		-0.312	**

KIRC, kidney renal clear cell carcinoma; LGG, Brain Lower Grade Glioma; LUAD, lung adenocarcinoma; UVM, uveal melanoma; TAM, tumor-associated macrophage; Th, T helper cell; Tfh, Follicular helper T cell; Treg, regulatory T cell; Cor, R value of Spearman's correlation; None, correlation without adjustment. Purity, correlation adjusted by purity. *p<**0.**05; **p<**0.**01; ***p<**0.**001; ****p<**0.**0001.

**Table 2:** Correlation analysis between CA4 and related genes and markers of immune cells in GEPIA.

Description	Gene markers	KIRC						LGG			LUAD						UVM	
		Tumor			Normal			Tumor			Tumor			Normal			Tumor	
		R	P		R	P		R	P		R	P		R	P		R	P
Monocyte	CD86	-0.17	****		0.23	0.051		-0.33	****		0.053	0.24		-0.19	0.15		-0.25	*
	CD115 (CSF1R)	-0.059	0.17		0.23	*		-0.28	****		0.079	0.082		-0.28	*		-0.15	0.2
TAM	CCL2	0.32	****		-0.24	*		-0.33	****		-0.014	0.76		0.0011	0.99		0.057	0.62
	CD68	-0.13	**		**0.4**	***		-0.38	****		0.18	****		-0.19	0.15		0.13	0.25
	IL10	-0.074	0.092		0.0092	0.94		-0.37	****		0.16	***		-0.35	**		-0.13	0.24
M1 Macrophage	INOS (NOS2)	**0.46**	****		0.068	0.57		0.27	****		0.3	****		**0.46**	***		0.038	0.74
	IRF5	-0.1	*		**-0.62**	****		-0.28	****		0.095	*		-0.28	*		-0.27	*
	COX2 (PTGS2)	0.051	0.24		-0.35	**		0.027	0.54		-0.088	0.052		-0.0043	0.97		-0.28	*
M2 Macrophage	CD163	-0.1	*		0.12	0.31		**-0.4**	****		0.14	**		-0.2	0.13		-0.17	0.14
	VSIG4	-0.12	**		0.15	0.21		-0.38	****		0.17	***		-0.25	0.061		-0.2	0.074
	MS4A4A	-0.069	0.12		0.26	*		-0.38	****		0.2	****		-0.24	0.071		-0.11	0.35
Th1	T-bet (TBX21)	0.19	****		0.36	**		-0.22	****		0.2	****		**0.43**	***		-0.34	**
	STAT4	-0.027	0.54		0.17	0.14		**0.41**	****		0.079	0.085		0.2	0.14		-0.19	0.1
	STAT1	-0.069	0.12		-0.29	*		-0.22	****		-0.015	0.73		0.11	0.41		-0.27	*
	IFN-γ (IFNG)	-0.24	****		0.02	0.87		-0.1	*		-0.0043	0.92		0.059	0.66		-0.25	*
	TNF-α (TNF)	0.046	0.29		-0.078	0.51		-0.15	***		0.02	0.66		-0.2	0.13		-0.33	**
Th2	GATA3	-0.021	0.64		**-0.41**	***		-0.23	****		0.013	0.77		0.39	**		-0.32	**
	STAT6	0.25	****		**-0.4**	***		0.052	0.24		0.28	****		0.19	0.15		-0.2	0.083
	STAT5A	-0.1	*		0.017	0.89		-0.26	****		0.13	**		-0.047	0.72		0.28	*
	IL13	0.12	**		-0.067	0.58		-0.018	0.68		0.069	0.13		-0.079	0.55		-0.34	**
Treg	FOXP3	-0.33	****		-0.16	0.18		0.22	****		-0.019	0.68		-0.17	0.19		-0.22	0.052
	CCR8	-0.22	****		0.21	0.074		-0.061	0.16		0.043	0.34		-0.15	0.26		-0.26	*
	STAT5B	**0.41**	****		-0.093	0.44		-0.18	****		0.23	****		**0.41**	**		0.015	0.9
	TGFβ (TGFB1)	-0.11	*		**-0.57**	****		-0.36	****		0.014	0.76		-0.05	0.7		-0.054	0.64
T cell exhaustion	PD-1 (PDCD1)	-0.2	****		0.39	***		-0.3	****		0.0068	0.88		-0.0078	0.95		-0.37	***
	CTLA4	-0.2	****		0.15	0.22		-0.1	*		0.054	0.23		-0.05	0.71		-0.29	**
	LAG3	-0.24	****		**-0.57**	****		-0.24	****		-0.055	0.22		0.072	0.59		-0.35	**
	TIM-3 (HAVCR2)	0.028	0.52		**0.59**	****		-0.33	****		0.076	0.095		-0.19	0.14		-0.22	*
	GZMB	0.089	*		0.35	**		-0.092	*		-0.047	0.3		**0.43**	***		-0.29	**

KIRC, kidney renal clear cell carcinoma; LGG, Brain Lower Grade Glioma; LUAD, lung adenocarcinoma; UVM, uveal melanoma; TAM, tumor-associated macrophage; Th, T helper cell; Treg, regulatory T cell; R, R value of Spearman's correlation. *p<**0.**05; **p<**0.**01; ***p<**0.**001; ****p<**0.**0001.

## Abbreviations

CA4: carbonic anhydrase 4
TCGA: the Cancer Genome Atlas
TISIDB: tumor-immune system interactions
OS: overall survival
PFS: progression-free survival
PPI: protein-protein interaction
STRING: search tool for the retrieval of interacting genes
TIMER: tumor immune estimation resource
GEPIA: gene expression profiling interactive analysis
CI: confidence interval
GO: gene ontology
BP: biological processes
CC: cellular components
MF: molecular functions
KEGG: Kyoto Encyclopedia of Genes and Genomes
KIRC: kidney renal clear cell carcinoma
LGG: brain lower grade glioma
LUAD: lung adenocarcinoma
PRAD: prostate adenocarcinoma
UVM: uveal melanoma
BLCA: bladder urothelial carcinoma
BRCA: breast invasive carcinoma
COAD: colon adenocarcinoma
ESCA: esophageal carcinoma
HNSC: head and neck squamous cell carcinoma
KICH: kidney chromophobe
KIRP: kidney renal papillary cell carcinoma
LUSC: lung squamous cell carcinoma
READ: rectum adenocarcinoma
STAD: stomach adenocarcinoma
THCA: thyroid carcinoma
UCEC: uterine corpus endometrial carcinoma
CHOL: cholangiocarcinoma
LIHC: liver hepatocellular carcinoma
SKCM: skin cutaneous melanoma
