# Supplementary material for: Carbonic Anhydrase 4 serves as a Clinicopathological Biomarker for Outcomes and Immune Infiltration in Renal Cell Carcinoma, Lower Grade Glioma, Lung Adenocarcinoma and Uveal Melanoma
Source: J Cancer. 2020 Aug 25;11(20):6101–13. doi: 10.7150/jca.46902 (PMC7477427; doi:10.7150/jca.46902)

Supplementary Figure 1: Univariate Cox regression analysis of PFS and OS of *CA4* in KIRC, LGG, LUAD and UVM patients from TCGA cohort.

(A-B) pT stage, pM stage, AJCC stage and ISUP grade significantly correlated to both PFS and OS in KIRC patients. Gender was significantly relevant to PFS while age and pN stage significantly correlated to OS in KIRC patients (p < 0.05). (C-D) Age and neoplasm grade significantly correlated to both PFS and OS and histological type significantly correlated to OS in LGG patients (p < 0.05). (E-F) pT stage, pN stage and AJCC stage significantly correlated to both PFS and OS and pM stage significantly correlated to OS in LUAD patients (p < 0.05. (G-H) Cell type was significantly relevant to both PFS and OS in UVM patients. Meanwhile, age and tumor basal diameter significantly correlated to OS in UVM patients (p < 0.05). Remarkably, CA4 amplification was obviously related to better PFS (KIRC: hazard ratio [HR] = 0.661, p < 0.001; LGG: HR = 0.552, p = 0.002; LUAD: HR = 0.922, p = 0.020; UVM: HR = 0.454, p = 0.001) and better OS (KIRC: HR = 0.847, p < 0.001; LGG: HR = 0.552, p < 0.001; LUAD: HR = 0.918, p = 0.007; UVM: HR = 0.454, p < 0.001) in all of these four cancers.

PFS, progression-free survival; OS, overall survival; KIRC, kidney renal clear cell carcinoma; LGG, brain lower grade glioma; LUAD, lung adenocarcinoma; UVM, uveal melanoma; TCGA, the cancer genome atlas.

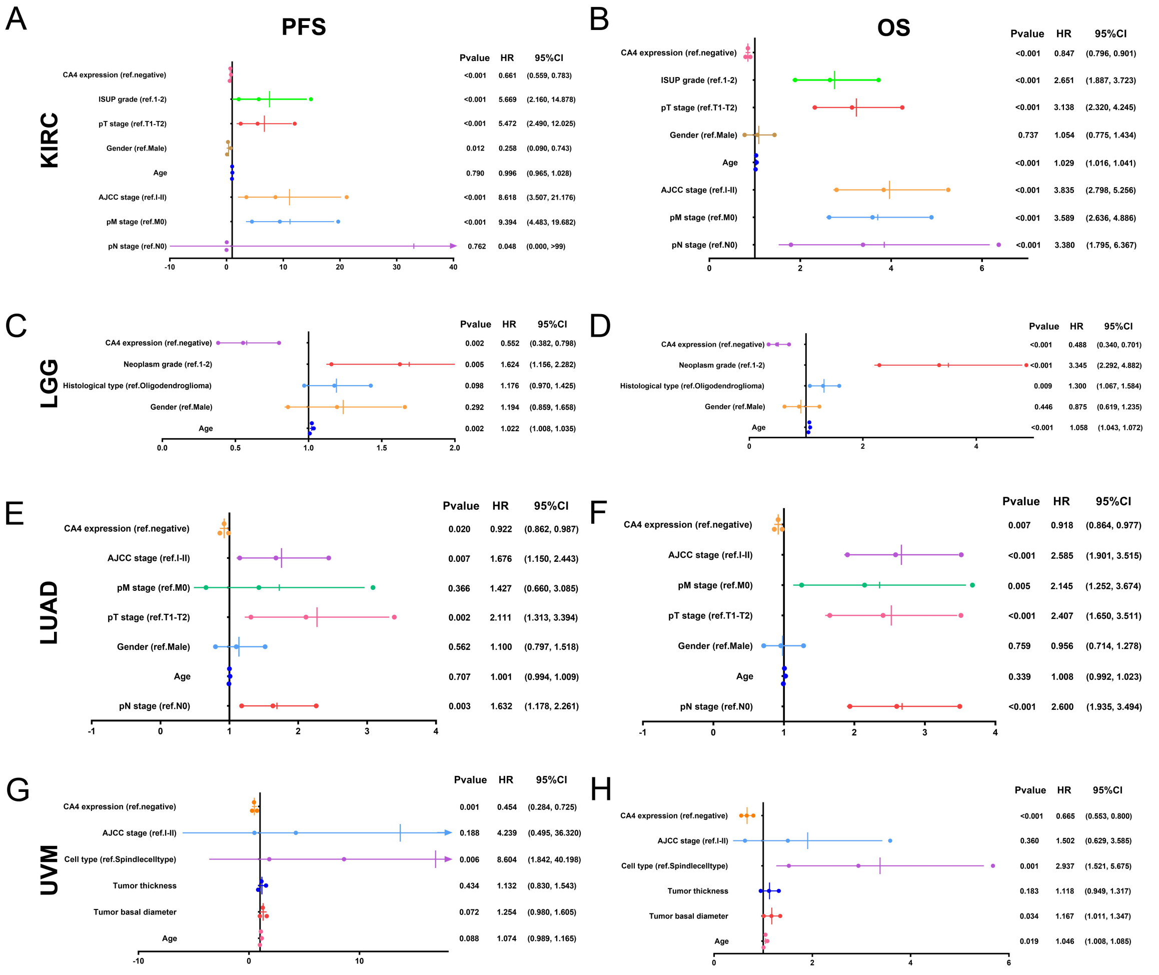

Supplement: Supplementary file 1 — Supplementary figure. [file jcav11p6101s1.pdf]
